# Effects of a Metabolic Mixture on Gut Inflammation and Permeability in Elderly Patients with Chronic Kidney Disease: A Proof-of-Concept Study

**DOI:** 10.3390/metabo12100987

**Published:** 2022-10-18

**Authors:** Roberto Aquilani, Piergiorgio Bolasco, Stefano Murtas, Roberto Maestri, Paolo Iadarola, Cristian Testa, Maria Luisa Deiana, Maria Paola Esposito, Rita Contu, Mariella Cadeddu, Romina Secci, Federica Boschi

**Affiliations:** 1 Department of Biology and Biotechnology “Lazzaro Spallanzani”, University of Pavia, 27100 Pavia, Italy; 2 Chronic Kidney Disease Study Group of Italian Society of Nephrology, 00185 Rome, Italy; 3 Territorial Department of Nephrology and Dialysis—ASL di Cagliari, 09125 Cagliari, Italy; 4 Department of Biomedical Engineering of the Montescano Institute, Istituti Clinici Scientifici Maugeri IRCCS, Montescano, 27040 Pavia, Italy; 5 Functional Point, Clinical and Virology Laboratory, 25121 Bergamo, Italy; 6 Department of Drug Sciences, University of Pavia, 27100 Pavia, Italy

**Keywords:** chronic kidney disease, gut inflammation and permeability, mitochondrial intermediates

## Abstract

Intestinal barrier dysfunction is a risk factor for the progression of Chronic Kidney Disease (CKD). In this proof-of-concept study, we tested the effects of a mixture of Essential Amino Acids (EAAs) and mitochondrial substrates on intestinal inflammation and permeability of CKD patients. Eight patients with stage 3b-4 CKD and 11 healthy controls after overnight fasting underwent fecal measures of calprotectin and zonulin levels (indicators of gut inflammation and permeability, respectively) and determinations of plasma amino acids. Only CKD patients were supplemented with the mixture (8 g/d diluted in water). Compared to controls, baseline fecal calprotectin, zonulin and plasma levels of some AA in CKD patients were significantly higher (*p* = 0.005; *p* = 0.001 and *p* = 0.02 to 0.003, respectively). After six months of supplementation, CKD baseline fecal levels of calprotectin and zonulin significantly (borderline for zonulin) decreased (*p* = 0.008 and *p* = 0.05, respectively). Plasma AA concentrations, including glutamine and alanine, were higher than at the baseline (*p*: 0.05 to 0.008). The supplementation of this mixture was associated with improved intestinal barrier dysfunction. Increased plasma AA levels might contribute to the improvement of gut barrier dysfunction.

## 1. Introduction

In Chronic Kidney Disease (CKD), a combination of gut inflammation and gut permeability [1,2,3] may cause intestinal barrier disruption, leading to translocations of lumen bacteria and toxic bacterial by-products (enterotoxins) into systemic circulation (endotoxemia). As documented both in animals and in humans with advanced CKD, endotoxemia is responsible for the development of systemic inflammation, acceleration of CKD progression, uremic syndrome, cardiovascular disease and an increased risk of mortality [4,5,6,7,8]. In CKD, gut dysbiosis, i.e., alterations of intestinal microbiota, plays a key role in gut inflammation [9]. For example, while in physiological conditions, duodenum and jejunum are not colonised by bacteria, in CKD, they are colonised by aerobic and anaerobic bacteria, which are responsible for a variety of chronic inflammations of the gastrointestinal tract such as esophagitis, gastritis, duodenitis, enteritis and colitis [10].

The main factors that cause gut dysbiosis are azotemia levels, dietary restriction and intestinal ammonia [1,11].

Increased intestinal permeability in patients with CKD follows the disruption and depletion of intestinal Tight Junction (TJ) structures caused by elevated azotemia [1,12], the presence of uremic toxins [13] and gut dysbiosis [11].

TJs are dynamic protein structures localized in the apical portion of enterocytes. They connect both epithelial and endothelial cells [14]. 

TJ proteins are comprised of [14] transmembrane proteins including Claudins, occludins, junctional adhesion molecules, tricellulin and angulins. All these proteins interact with intracellular scaffolding proteins (Zonula Occludens, Zos). The integrity of TJs derives from the integrity of their connections.

Since TJs serve to control the transition of molecules through the paracellular space [14], their disruption and depletion lead to loss of barrier homeostasis and increased intestinal permeability.

Gut inflammation and permeability represent two mutually conditioning factors, in that inflammation with the presence of cytokines [15,16] disrupts TJs and, in turn, dysregulated TJs permit intestinal translocation with activation of both local and systemic inflammation. Only in the presence of TJ alterations can bacteria and bacterial by-products enter systemic circulation [1].

For clinical purposes, the measurement of elevated fecal calprotectin and Zonulin (Zo) can be used for the diagnosis of intestinal inflammation [17] and permeability [18], respectively.

Zo belongs to the Zo family of TJ proteins and interacts with claudin-Z. Zo is the only human protein that modulates intercellular TJs to reversibly regulate intestinal permeability [14,18].

Zo release is triggered by bacteria dysbiosis and gliadin [14,18]. Abnormal secretion of Zo has been found in diabetes, celiac disease, tumors [18,19] and pediatric patients with inflammatory bowel disease [20].

Based on all the above knowledge, in this proof-of-concept study, we hypothesized that chronic supplementation (6 months) with a mixture containing Essential Amino Acids (EAAs) and some mitochondrial intermediates could reduce both intestinal inflammation and permeability in patients with moderate to severe CKD. The rationale behind the hypothesis is as follows. First, Amino Acids (AAs) are essential in the intestine [21] to maintain its integrity. The human intestine, in fact, uses 20% to 80% of dietary EAAs [22,23,24,25,26,27,28,29,30]. Second, EAAs are the building blocks of protein synthesis. Since TJ proteins have a fast turnover [31,32], they require adequate AA intake (in particular EAAs) in the intestinal lumen. Third, EAAs may exert an anti-inflammatory activity [33,34,35]. Fourth, in an experimental model of CKD (in mice), supplemented essential Branched-Chain AAs (BCAAs) have shown their capacity to re-equilibrate altered intestinal microbiota towards a development of healthier strains [36]. 

Fifth, mitochondrial intermediates, in addition to generating aerobic energy, can act as regulators of the immune response [37]. 

The ultimate intent of this study was to highlight the possible role of amino acids in gut barrier dysfunction, thus providing a plausible pathophysiological background for future investigations.

## 2. Methods

This experimental study relies on the same population of CKD patients and healthy subjects (controls: CTRL) who took part in a previously described observational study (submitted to Clinical Nutrition), in which plasma Amino Acid (AA) levels and fecal calprotectin and Zo contents were monitored at enrolment and after 12 months. These patients were asked to undergo 6 months of supplementation with a mixture containing Essential Amino Acids (EAAs) and some intermediates of Tricarboxylic Acid (TCA) cycle. Measures taken after 12 months of survey served as baseline values for the current proof-of-concept study.

Twenty-nine non-smoking elderly (age > 65 years) patients had been preselected for having a diagnosis of stage 3b-4CKD (estimated glomerular filtration-eGFR- rate from 44–29 mL/min/1.73 m^2^). The reason for choosing elderly subjects was that it would be easier to find alterations in gut barrier integrity, as ageing is associated with loss of Tight Junctions (TJ), microbial dysbiosis and intestinal barrier dysfunction [31]. The reason for selecting non-smoking patients was that cigarette smoking is associated with altered intestinal TJ [38]. Patients with concomitant acute and chronic inflammatory diseases (*n* = 3), diabetes (*n* = 5), cancer (*n* = 1), autoimmune diseases (*n* = 1), chronic obstructive pulmonary diseases (*n* = 7), heart failure (*n* = 2) and liver diseases (*n* = 1) and patients on steroid/glucocorticoids/mineralocorticoids immunosuppressant treatment (*n* = 1) were excluded from the study. Moreover, patients with eGFR >44 and <15 mL/min/17.3 m^2^ and healthy volunteers with eGFR <80 mL/min/1.73 m^2^ were excluded from the study. Therefore, a total of 8 out of the initial 29 patients were investigated. 

The patients were on a diet treatment with protein intakes of 0.6–0.7 g/kg (50% of animal origin), caloric intake >30 Kcal/kg/day and phosphate intake <1000 mg/day.

In addition to eGFR, renal function was evaluated by means of the prediction Modification of Diet in Renal Disease study group (MDRD, ml/min/1.37 sqm) [38] that was normalized to the body surface area.

After enrolment, the patients underwent the following measurements:

1. Biohumoral variables, including 24 h urine protein content. Urea Nitrogen Appearance (UNA) was calculated to estimate protein intake [39].
2. Body composition: Total Body Water (TBW), Extracellular Water (ECW) and Intracellular Water (ICW), all in liters (L), percentage of body weight, Resistance (RZ in Ohm), Conductance (XC in Ohm) and phase angle (in degrees), considered an indicator of skeletal muscle mass [40], were measured by Bioelectrical Impedance Analysis (BIA) by the same operator who used the same instrument (renal EFG 50 Hz,; EFG Diagnostic Ltd., Belfast, Northern Ireland).
3. Determination of plasma AAs.

At 8 a.m., after 12 h of overnight fasting, blood samples were drawn from patients through antecubital veins and immediately delivered to the laboratory, where plasma was obtained from heparinized blood using centrifugation (800× *g*, 15 min). The concentration of free amino acids in the plasma was measured using an AminoQuant II amino acid analyser, based on the HP 1090 HPLC system, with fully automated precolumn derivatization. Both orthopthalaldehyde (OPA) and 9-fluorenyl-methyl-chloroformate (FMOC) reaction chemistries were used, according to the manufacturer’s protocol. Measurements were made by injecting 1 µL of the derivatized mixture and measuring absorbance simultaneously at 338 and 262 nm [41]. Plasma concentrations were expressed as µmol/L. The measurements of the plasma amino acids were carried out in triplicate by the same laboratory. The mean of the three measurements was calculated and adopted. The characteristics of the method were based both on precision and standardization properties (unpublished data): based on precision, relative standard deviation (RSD) was 1.13%; reliability (bias) was 10.37%; the lower limit of quantitation was 0.18 ng/mL; the limit of detection was 0.6 ng/mL. For the measurements in triplicate, the intra-day variability (RSD) was 3.21%, and the intervariability was 4.77%. The AAs measured were as follows: (1) Essential AAs (EAAs) included leucine, valine, isoleucine, lysine, threonine, phenylalanine, methionine and tryptophan. The first three EAAs constitute the Branched-Chain AAs (BCAAs). (2) Non-EAAs included aspartic acid, glutamic acid, histidine, asparagine, serine, glutamine, arginine, citrulline, glycine, alanine, taurine and tyrosine.

4. Determination of fecal calprotectin and Zo levels.

Calprotectin determination in stool samples was performed by an immunoenzymatic method and measured by Chorus TRIO instrument (DIESSE Diagnostica Senese S.p:A., Siena, Italy) according to the manufacturer’s instructions. Calprotectin values <50 µg/g per stool sample were considered normal. 

Intestinal permeability was evaluated as a fecal Zo concentration (ng/mL) using a commercial ELISA kit (Zonulin Stool ELISA, DRG Instruments Gmbh, Heppenheim, Germany). The normal amount of Zo in feces of healthy subjects is considered to be <60 ng/mL.

Eleven elderly, healthy, non-smoking subjects, matched for age, sex and Body Mass Index (BMI, Kg/m^2^) served as CTRLs.

The CTRLs underwent the same procedures as CKD patients but only at the time of their enrolment. 

After completing the above evaluations, only patients were prescribed a mixture containing AAs + 3 intermediates of mitochondrial TCA cycle (citric acid, succinic acid, malic acid) (Amino-Ther Pro^®^ Professional Dietetics, Milano, Italy) (Table 1). The patients were instructed to take 2 packets/day for 6 months (1 in the morning and 1 in the afternoon, diluted in 150–200 mL of water). The reasons for choosing this type of mixture were as follows: (1) the documented efficacy of this combination on the enhancement of oxidative metabolism, on mitochondrial function, and on the prevention of oxidative stress [42,43], in addition to the documented effects of EAAs on improving protein synthesis both in animals [44] and humans, including those who are healthy [45] and those affected by chronic disease [34,46,47]; (2) some mitochondrial TCA intermediates (including those contained in the mixture) act as pro/anti-inflammatory agents [48,49].

All the above procedures were repeated in CKD patients after 6 months of mixture supplementation.

5. Evaluation of patient adherence to the mixture.

Patients were considered adherent to the prescribed mixture if, after overnight fasting, at 6 months from starting supplementation, their plasma glutamine and alanine had significantly increased in relation to baseline values. 

Increased plasma glutamine and alanine levels should follow increased muscle utilization of Branched Chain Amino Acid (BCAA; contained in the mixture). Moreover, to confirm the BCAA-induced glutamine and alanine formations, we added an increase in plasma levels of two ratios: glutamine/(BCAA + aspartic acid + asparagine + glutamic acid) and alanine/(BCAA + aspartic acid + asparagine + glutamic acid). Indeed, aspartic acid, asparagine and glutamic acid also contribute to glutamine [50] and alanine [51] formations.

6. Estimation of the effects of the mixture on ureagenesis.

It may be especially important in the case of progression of GFR to determine whether a possible increase in BUN was due to increased nitrogen intake as a result of mixture supplementation and/or to worsened renal dysfunction. Following our reasoning, non-increased or reduced BUN/plasma total AA ratio in fasting patients points to the absence of nitrogen intake overload. 

In summary, in the current study, we compared (1) the differences in the measured variables between CKD patients before supplementation and the CTRL group and (2) the changes in variables of the CKD patients at baseline and 6 months after mixture supplementation. 

## 3. Statistical Analysis

Central tendency and dispersion of continuous variables are reported as mean ± standard deviation. Discrete variables are reported as numbers (N) and frequency percentage. Several variables violated the normality assumption (Shapiro–Wilk test); hence, non-parametric statistics were preferred for hypothesis testing. Within- and between-group comparisons for continuous variables were carried out by the Wilcoxon signed rank test and by the Mann–Whitney U-test, respectively. Categorical variables were compared by the Chi-square test or Fisher’s exact test if appropriate. The association between couples of variables was assessed using Spearman’s correlation coefficient.

All tests were two-tailed. A *p* value < 0.05 was considered statistically significant.

When appropriate, false discovery rate was controlled at 5% (Benjamini–Hochberg method).

All statistical analyses were carried out using the SAS/STAT statistical package, release 9.4 (SAS Institute Inc., Cary, NC, USA).

## 4. Objectives of the Study

The primary objective of the study was to reduce fecal calprotectin and Zo levels, and the secondary objective was to improve patients’ phase angle and renal function.

The study was approved by the Ethics Committee of the Local Health Authorities (Reg 74/2018) after the entire population provided their written informed consent. 

## 5. Results

### 5.1. Patients’ Baseline Characteristics vs. Controls (CTRL) (Table 2)

CKD patients and CTRLs had similar ages and body weight (as BMI) but different body compositions. Indeed, the patients showed increased Total Body Water (TBW; *p* < 0.012) and Extracellular Body Water (EBW); *p* < 0.0001) and reduced phase angle (*p* = 0.008), which is considered an indicator of a decreased skeletal muscle mass [40].

When compared to CTRLs, CKD patients showed mild anemia with lower serum levels of iron (*p* = 0.005) and ferritin (*p* = 0.016), whose mean values were still within the normal range of our laboratory.

From a renal standpoint, CKD patients exhibited moderate to severe renal failure (MDRD 30.83 ± 7.88 mL/min/1.73 sqm) and compensated metabolic acidosis (reduced plasma bicarbonate levels, *p* < 0.01) compared to CTRLs.

Blood Urea Nitrogen (BUN) was higher in CKD patients than in CTRLs (*p* < 0.001), whereas protein intake from UNA was lower (*p* < 0.05) but in absolute value higher than that prescribed (0.6–0.7 g/kg/d).

**Table 2:** Baseline characteristics of controls (CTR) and Chronic Kidney Disease (CKD) patients.

Variables	CTR	CKD	*p*
**Demographic, antrophometric variables**			
Male gender n (%)	4 (36%)	4 (50%)	0.66 *
Age (years)	72.27 ± 3.74	74.56 ± 6.90	0.32
Body Weight (Kg)	64.50 ± 12.80	62.76 ± 9.05	0.61
Body surface (sqm)	1.72 ± 0.20	1.66 ± 0.15	0.40
Body mass index (Kg/m^2^)	24.86± 2.79	25.31 ± 2.31	0.50
**BIA measures**			
Resistance (Ohm)	536.3 ± 40.0	515.5 ± 113.2	0.99
Conductance (Ohm)	52.15 ± 12.42	42.25 ± 17.38	0.17
Phase angle (NV > 5.0 degrees A°)	5.16 ± 0.60	4.22 ± 0.67	0.008 ^
Total Body Water (%)	52.15 ± 12.42	55.93 ± 9.20	0.012 ^
Extracellular Body Water (%)	41.61 ± 15.61	55.75 ± 4.39	<0.0001 ^
Intracellular Water (%)	35.65 ± 16.6	40.69 ± 10.06	0.59
**Biohumoral variables**			
Modification of Diet in Renal Disease	88.20 ± 16.59	30.83 ± 7.88	<0.0001 ^
Glucose (NV. 70–100 mg/dL)	94.36 ± 8.03	97.22 ±12.43	0.45
Blood Urea Nitrogen (NV 9.35 ± 18.7 mg/dL)	11.47 ± 3.52	43.86 ± 16.09	<0.0001 ^
Hemoglobin (NV F > 12; M > 13 g/dL)	14.16 ± 1.20	11.85 ± 1.12	0.0006 ^
Iron (NV 40–160 ug/dL)	106.09 ± 26.69	72.38 ± 32.76	0.005 ^
Ferritin (NV 30–230 ng/mL)	175.60 ± 93.28	78.97 ± 60.65	0.016 ^
Calcium (NV 8.2–10 mg/dL)	9.72 ± 0.58	9.41 ± 0.52	0.14
Phosphorus (NV 2.8–4.1 mg/dL)	3.54 ± 0.60	4.14 ± 0.90	0.046
Albumin (NV 4.0–4.8 g/dL)	4.19 ± 0.20	6.94 ± 0.45	0.45
Total protein (NV 6.2–8.0 g/dL)	7.21 ± 0.043	7.07 ± 0.57	0.71
Sodium (NV 136–146 mEq/L)	141.03 ± 2.12	141.14 ± 2.89	0.49
Potassium (NV 3.5–5.1 mEq/L) Triglycerides (NV 50–150mg/dl) Total cholesterol (NV 100–200 mg/dl) HDL cholesterol (NV > 40 mg/dl) LDL cholesterol (NV 0–100 mg/dl) Reactive C-protein (NV > 0.8 mg/dl)	4.31 ± 0.41 98.91 ± 69.06 222.27 ± 23.09 64.18 ± 18.87 137.09 ± 22.52 0.13 ± 0.19	4.36 ± 0.47 75.50 ± 19.57 193.13 ± 34.69 77.86 ± 32.19 102.50 ± 20.79 0.30 ± 0.54	0.79 0.21 0.04 0.3 0.009 0.2
**Kidney function**			
24 h urine proteins (NV <100)	109.11 ± 78.02	324.04 ± 316.12	0.21
Plasma creatinine (NV 0.6–1.4 mg/dL)	0.8 ± 0.16	3.25 ± 1.43	<0.0001 ^
Estimated Glomerular Filtration Rate (eGFR; ml/min/1.73 sqm)	88.1 ±19.63	27.79 ± 3.00	<0.0001 ^
Modification of Diet in Renal Disease (MDRD, ml/min/1.73 sqm)	88.20 ± 16.59	30.83 ± 7.88	<0.0001 ^
Urinary nitrogen appearance (UNA, protein g/kg/day)	1.08 ± 0.21	0.89 ± 0.21	0.02 ^
**Blood acid-base status**			
pH (NV 7.36–7.42)	7.36 ± 0.02	7.35 ± 0.04	0.4
Bicarbonate (NV 24–26 mEq/L	26.69 ± 2.18	23.03 ± 1.42	0.001 ^

Reported *p*-values are from Mann-Whitney U-test or * Fisher's Exact Test; NV: normal value ^: significance confirmed controlling for the False detection rate at 5% (Benjamini Hockberg method.

**Figure 1:**
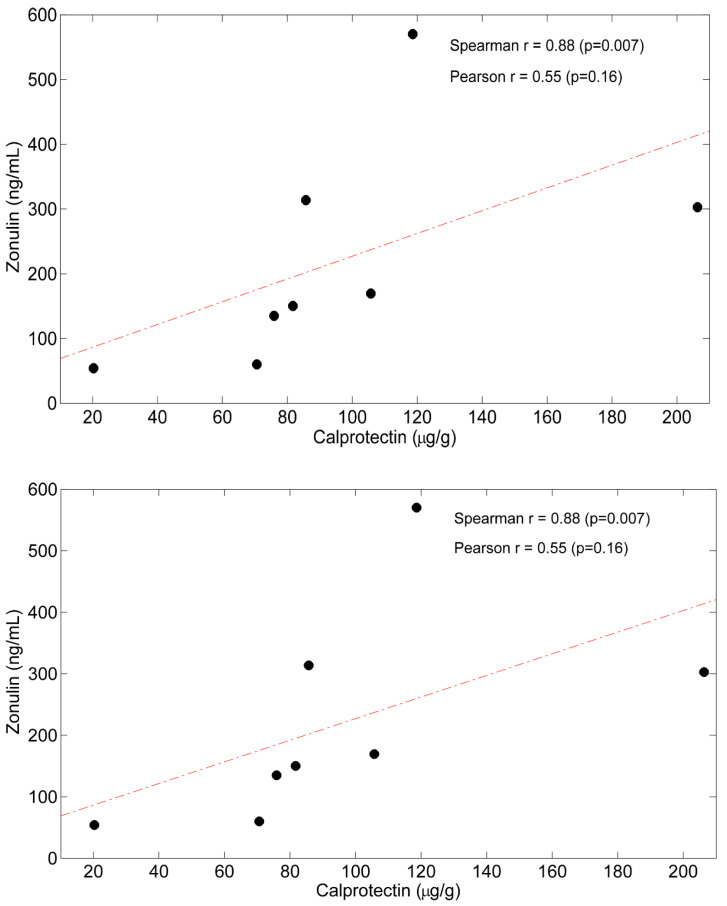
Scatterplot showing the association between baseline fecal Calprotectin and Zonulin. Spearman’s r and Pearson’s r are also reported. The red dash-dotted line represents the linear fit.

With respect to the intestine (Table 3), CKD patients showed a state of intestinal mucosa inflammation (higher fecal calprotectin, *p* = 0.005 vs. controls) and higher permeability (higher fecal Zo concentrations, *p* < 0.001 vs. controls). The correlation between fecal calprotectin and fecal Zo was significant (r +0.88, *p* < 0.007) (Figure 1).

With respect to the plasma AA profile (Table 4), compared to CTRLs, CKD patients had higher levels of aspartic acid (*p* = 0.0005), asparagine (*p* = 0.034), serine (*p* = 0.016), histidine (*p* = 0.002) and glycine (*p* = 0.043) and higher phenylalanine/tyrosine ratio (phe/tyr ratio; *p* = 0.01). 

CKD patients and CTRLs had similar plasma levels of Total Amino Acids (TAAs), EAAs, non-EAAs and BCAAs. 

### 5.2. Changes in Baseline Variables after 6 Months of EAA Supplementation (T_6_-Baseline)

After EAA supplementation, a progression of renal filtration damage (reduced MDRD, from baseline 30.83 ± 788 vs. 25.92 ± 9.02 mL/min/1.73 sqm; *p* = 0.039) and increased proteinuria (*p* < 0.008) occurred. On the contrary, fecal levels of calprotectin and Zo significantly decreased (−60% and −68%, respectively, borderline significant for Zo, in comparison to baseline values), indicating reduced intestinal inflammation and permeability (Table 5).

Supplemented EAAs were also associated with changes in plasma levels of several AAs (Table 6): aspartic acid (*p* = 0.016), asparagine (*p* = 0.016), serine (*p* = 0.016), glutamine (*p* = 0.008), threonine (*p* = 0.05), alanine (*p* = 0.023) and methionine (*p* = 0.008). NON-EAAs (*p* = 0.05) increased, whereas glutamic acid decreased (*p* = 0.016). Moreover, the phe/tyr ratio was higher than that at baseline (*p* = 0.05).

### 5.3. Correlations between Time Courses of Calprotectin, Zo and Plasma AAs

The changes over time in calprotectin did not correlate with the time courses of plasma AAs. On the contrary, the reduction in fecal Zo was positively associated with the increase in plasma EAAs (r = +0.857, *p* = 0.024) and valine (r = +0.714, *p* = 0.05) (Table 7).

### 5.4. Estimation of Patients’ Adherence to EAA Prescription

The CKD patients had a good adherence to the prescribed EAA supplementation, as shown by the increased glutamine/(BCAA + aspartic acid + asparagine + glutamate) ratio (*p* = 0.008) and the alanine/(BCAA + aspartic acid + asparagine + glutamate) ratio (*p* = 0.008) (Table 8).

### 5.5. Estimation of Supplemented Mixture Effect on Ureagenesis

The study showed that despite the progression of renal damage, circulating urea levels did not increase (Table 9). This means that supplemented EAAs did not give rise to significant urea generation, as also shown by BUN/Tot AAs similar to baseline value (Table 9).

## 6. Discussion

The study shows that elderly patients with CKD at stage 3b-4 have reduced gut integrity because of inflammation and TJ depletion, which increases intestinal permeability [14]. Moreover, the results indicate good patient adherence to EAA supplementation, which was associated with improved gut inflammation and permeability and changes in several plasma AA levels. There was a positive correlation between improvements of gut inflammation and TJ depletion, on one hand, and the increase in plasma EAAs and valine, on the other hand.

The improvement in gut integrity reduces the risks of intestinal translocations [1,52,53]. Although correlations do not necessarily represent a cause/effect relationship, the levels of circulating EAAs and valine seem to play a crucial role in the repair and synthesis of TJ structures. The reasons are that EAAs are of paramount importance for protein synthesis, and valine, which occupies the terminal position of claudin-2 proteins, binds these proteins to zonula occludens protein [54].

Therefore, reduced plasma EAA availability (including valine) may lead to increased risk for loss of intestinal mucosa integrity. This is supported by a recent study carried out in mice that documents how a diet with an excess in the NEAA/EAA ratio of the nitrogen content (overrepresented NEAAs with respect to EAAs) caused a reduction in intestinal cell Zo content and an increase in fecal excretion, indicating increased intestinal permeability [55].

In addition to their effects on TJ structures, EAAs may reduce intestinal inflammation, as AAs may exert anti-inflammatory effects [33,35,56]. In elderly diabetic patients, supplemented EAAs decrease serum TNF α levels [34,47]. Therefore, ingested EAAs in the intestinal lumen of the study patients could reasonably act as anti-inflammatory agents. 

Although gut inflammation, TJ depletions and plasma AAs are mutually influencing factors, they will be discussed separately for more clarity.

### 6.1. Gut Inflammation at Baseline and 6 Months after EAA Supplementation

In the current study, gut dysbiosis was likely the main mechanism for gut inflammation. In CKD, uremia, intestinal ammonia and dietary restriction are the main causes of pathogenic gut microbiota [9]. 

Gut dysbiosis activates host proinflammatory pathways, which are crucial for gut barrier dysfunction [57].

In animal CKD models, uremia was shown to reduce intestinal microbiota diversity and intestinal barrier function [6] and favor the development of gut bacteria capable of proteolytic fermentation [9]. The latter is responsible for the production of proinflammatory enterotoxins such as indoxylsulfate from dietary tryptophan and p-cresyl sulfate from dietary tyrosine. Translocated enterotoxins across the disrupted intestinal barrier invade the systemic circulation and accumulate in systemic circulation (endotoxemia). If they are not cleared because of renal dysfunction, they cause a progression of kidney alterations, cardiovascular disease and systemic inflammation [4]. Importantly, bacterial endotoxemia appears at an early stage of CKD (stage 2) and increases to stage 5 [58].

Trimethylamine-N-oxidase, a by-product of bacteria that metabolize dietary choline and carnitine, and an advanced glycation end-product [13] are other potent proinflammatory and proatherogenic substances.

The changes in intestinal microbiota occur not only in the colon lumen [9] but also in the small bowel tract, such as the duodenum and jejunum, which are normally not colonized and become part of both aerobic and anaerobic bacteria development [10].

Ammonia, formed from the influx of plasma urea into the intestinal lumen, is another important factor for gut dysbiosis [59]. Intestinal urea indeed promotes the development of urease-forming bacteria, leading to ammonia formation [9,60], which causes mucosa irritation, enterocolitis and elevation of lumen pH [9,60].

Dietary restriction itself causes gut dysbiosis. Potassium restriction is associated with reductions in insoluble fiber intakes [60] and consequently with reductions of gut flora producing anti-inflammatory Short-Chain Fatty Acids (SCFA) [61].

Several investigations have documented the effects of EAAs in reshaping gut microbiota towards the development of healthier, anti-inflammatory bacteria. In addition, in-mixture threonine and EAA-induced increases in plasma glutamine and threonine promote the production and maintenance of the intestinal mucous layer. These may be among the main mechanisms that explain the improved gut inflammation in CKD patients. 

BCAAs, contained in the mixture used for this study, can induce the development and proliferation of healthier microorganisms [36], for example, by increasing the firmicutes/bacteroidetes ratio and diminishing the proteobacteria such as pathobiont enterobacteria [62].

Another pathogenic bacteria population that are reduced by lumen BCAA are the bacilli, including pathogenic streptococci, staphylococci and enterococci, which colonize the duodenum and jejunum in CKD patients [10]. On the contrary, BCAAs increase the akkermansia species that attenuate intestinal inflammation via induced intestinal endocannabinoid secretion [63]. Moreover, BCAAs induce the proliferation of health-promoting bifidobacterial species [36].

BCAAs may reduce gut inflammation by promoting the colonic development of bacteria producing SCFA, substrates with anti-inflammatory activities [64]. The SCFA butyrate and propionate induce, respectively, the differentiation and the maintenance of Treg cells.

BCAA supplementation significantly reduced serum levels of lipopolysaccharide-binding protein, an acute-phase protein that links lipopolysaccharide (LPS) and hence reflects the gut antigen load that translocates into the bloodstream [36].

The EAA threonine exhibits an immune anti-inflammatory activity, as a large proportion of this AA is absorbed by the gut for the production of intestinal immunoglobulins [65]. Moreover, threonine is of utmost importance for intestinal mucin synthesis, as it is a major component of secretory mucins [66,67,68,69].

Mucins are the main proteins of the mucus layer, which protects intestinal epithelial cells from lumen pathogens and toxic agents [70]. Loss of mucin reduces the protection of the mucus layer and leads to inflammation, loss of epithelial integrity and increased infiltrating cells [71], as secretory mucins are important for the innate immune defense of the mucosa [72]. Deficit of dietary threonine reduces gut mucin production [73]. Threonine, together with valine, alanine and aspartic acid [74], is produced by the bifidobacteria. Bacteria use threonine to form the EAA isoleucine.

Glutamine exerts potent immune anti-inflammatory activity on the intestinal mucosae. Although this AA was not contained in the supplemented mixture, its plasma levels increased after EAA supplementation. Glutamine is taken up by the intestinal mucosae cells [75,76], where, together with dietary glutamine, it exerts pro-energetic, immune- and biosynthesis activities. 

Indeed, glutamine is an important mitochondrial substrate [77] and decreases both local and systemic Interleukine-6 (IL-6), thus reducing local and systemic inflammation. Moreover, glutamine suppresses cytokine expression of IL-17 produced by T_H_ 17 and T_H_1 cells and other leukocyte subsets [78]. Il-17 disarrays TJs and increases the permeability of the small intestinal barrier [79,80]. Glutamine also exerts its beneficial effects by increasing γδ T cell activation, promoting T cell proliferation and protecting T cells against apoptosis [78].

The three mitochondrial substrates of the study mixture probably played a role in modulating the intestinal inflammatory pathways, given that TCA intermediates are also known to work as regulators of immune responses [37]. The modulation of inflammatory signaling by TCA intermediates is complex. Indeed, while citric acid, contained in the mixture, is proinflammatory via the activation of macrophages and dendritic cells [81], its by product cis-aconitate is the precursor of itaconate, a molecule that is upregulated upon immune activation and is a potent immunosuppressant agent [82,83]. Moreover, α-ketoglutarate, formed by iso-citrate, a by-product of citrate, and by glutamine and glutamate [84,85], deactivates proinflammatory macrophages [86,87] and induces anti-inflammatory macrophage [86,88] phenotype.

Succinic acid, contained in the study formula, promotes immune activation [88,89] and exacerbates inflammation [90], but its derived fumaric acid (not contained in the mixture) acts as an anti-inflammatory agent, given that fumarate degradation promotes recurrent infections [48].

Malic acid, contained in the mixture of this study, might counteract inflammation, given that its secretion from microbial cells is increased under lipopolysaccharide-induced macrophage activation [49]. Of note, oxidative phosphorylation and fatty acid oxidation are crucial to fuel immunosurveillance of T memory cells and immunosuppression of regulatory T cells, respectively [91,92,93].

In summary, given that the study shows a reduction in intestinal inflammation, we postulate that the mitochondrial intermediates of the mixture promoted a more balanced inflammatory/adaptive immune response towards adaptive immunity, acting synergically with EAAs.

### 6.2. Gut Permeability at Baseline and after 6 Months of EAA Supplementation

In the study patients, gut dysbiosis was likely the main cause of TJ disruption [1], as bacteria (other than gliadin) is the main stimulus for Zo secretion [9,14,94]. In the study, CKD development of intestinal proinflammatory bacteria may explain the observed correlation between fecal calprotectin and fecal Zo. That Zo reflects intestinal permeability has been also demonstrated in patients with ankylosing spondylitis [95], in whom serum Zo levels positively correlated with increased lactulose/mannitol ratio, whereas this was not the case for the control group.

The association of improved TJ abundance with EAA supplementation may be due to EAAs directly promoting TJ protein synthesis and indirectly stimulating the development of intestinal healthier bacteria that can contribute to both amino acid homeostasis of the host [96] and an increase in intestinal availability in AAs. Indeed, intestinal microorganisms including bifidobacteria can produce amino acids from dietary and endogenous fermentable carbohydrates [97,98]. Bacteria can produce the EAAs lysine and threonine [99] and produce isoleucine from threonine, as well as from non-specific nitrogen sources such as urea and ammonium chloride. Up to 21% of circulating lysine and 17% of threonine are produced by bacteria [100]. Dietary and bacterial amino acids are used in intestinal epithelial cells to sustain the synthesis of both intracellular protein and other energy-producing compounds including glutamate and glutamine [72]. At least 50% of these amino acids in the gut are completely oxidized to CO_2_. This accounts for the intense use of dietary and microbial amino acids by the intestine [72]

Among the EAAs in the mixture, tryptophan plays a pivotal role by regulating intracellular protein turnover of small intestine epithelial cells and the expression of TJ proteins in these cells [75]. Moreover, tryptophan modulates gastrointestinal motility, as it is the precursor of serotonin [101,102,103]. Small intestine mucosa has a substantial utilization of BCAA, as it possesses transaminase and BCAA-α ketoacid dehydrogenase [104]. Moreover, 20% of the ingested lysine, methionine, phenylalanine and threonine are estimated to be used by the intestine for mucosal protein synthesis [66].

It should be underlined, in particular for CKD subjects, that the intense use of amino acids by the intestinal mucosa may explain why 30–50% of dietary amino acids are not available to extraintestinal tissues [26].

In the study patients, the retrieval of gut TJ abundance may also be attributed to the plasma amino acid glutamine. Glutamine was not contained in the mixture but increased in plasma after EAA supplementation. Glutamine enhances TJ proteins in the small intestines of piglets [75] and gut integrity [76]. In-mixture succinic acid may contribute to improvement in gut permeability. In addition to being important for aerobic energy production [105], succinic acid can increase the abundance of TJ cloudin-1, ZO-1 and ZO-2 in the jejunum [106]. Therefore, succinic acid modulates intestinal epithelial barrier function [106].

**Figure 2:**
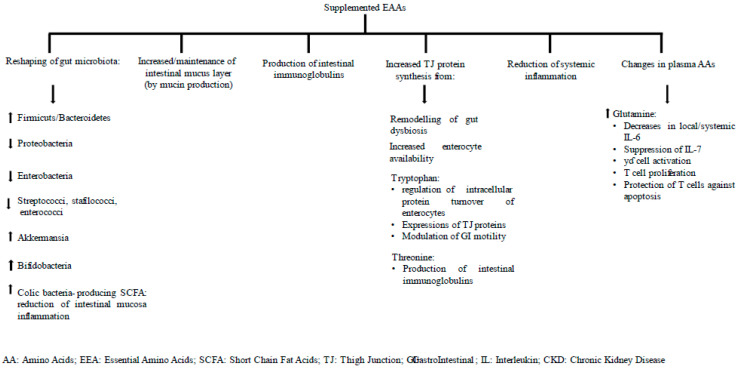
Some potential mechanisms underlying EAA-associated improved intestinal barrier dysfunction in the study CKD.

Figure 2 summarizes some mechanisms that underlie the positive effects of supplemented EAAs on intestinal barrier dysfunction.

### 6.3. Plasma Amino Acids at Baseline and after 6 Months of EAA Supplementation

At baseline, in comparison to CTRL, CKD patients had higher plasma levels of aspartic acid, asparagine, serine, histidine and phenylalanine. These alterations may indicate both renal and muscle dysmetabolism. Indeed, serine is exclusively produced by the kidney [107,108,109].

The higher plasma levels of phenylalanine were likely due to lack of liver conversion of the AA to tyrosine [107,108,110]. The deficit of phenylalanine conversion to tyrosine may explain the higher phenylalanine/tyrosine ratio in CKD than in CTRL.

Both aspartic acid and its derivative asparagine could derive from reduced availability of oxalacetate secondary to oxalacetate utilization for serine synthesis [109].

Increased production of Reactive Oxygen Species (ROS) following mitochondrial dysfunction [111] may lead to increased utilization of anserine and carnosine, two histidine-based dipeptides [112], which potentially explains the increased circulating histidine in the treated population.

Four aspects are evident from the altered circulating AAs after EAA supplementation. First, the baseline alterations of AAs were even more amplified. Second, significant increases in glutamine, alanine and methionine and a significant decrease in glutamate occurred. Third, with the exceptions of methionine and threonine, the altered AAs were not contained in the supplemented mixture. Fourth, with the exceptions of methionine and threonine, no other circulating EAAs underwent a significant change in the plasma. 

Likely, in-mixture BCAA increased muscle formation and release of glutamine and alanine via transamination activities [113]. Similarly, the changes in the other non-EAAs were likely an expression of increased transamination activities and not of muscle hypercatabolism, given that plasma phenylalanine levels were unchanged [114]. Indeed, for non-EAA formation, the amino group (-NH_2_) derives from glutamate transamination, whereas the carbon skeleton, in the final analysis, derives from α-ketoglutarate (α-KG), an important intermediate of the mitochondrial citric acid cycle. The muscle transamination hyperactivities are in line with an early study that documented hyperactivities of the transamination reactions in biopsied quadriceps of CKD patients [115]. Therefore, EAA supplementation seemed to increase muscle mitochondrial citric acid cycle activity. On the other hand, the three substrates contained in the mixture act as intermediates of the citric acid cycle and enhance mitochondrial function, explaining, in this way, the post-EAA amplifications [42]. Augmentation of citric acid cycle activity may explain the post-EAA amplifications of baseline altered AAs.

The absence of increases in circulating EAAs, except methionine and threonine, and the good patient adherence to EAAs suggest increased EAA utilizations by muscle and extramuscular districts. 

The increased methionine observed in the patients with CKD [116] may derive from the suppression of transmethylation reactions by S-Adenosyl-Homocysteine (SAH) [117]. SAH is both a potent inhibitor of intracellular methylation reactions and a factor that decreases eGFR [118]. It is not possible to exclude the possibility that supplemented methionine may contribute to the increased circulating levels, even though patients only ingested a minimal amount (discussed below). 

The alterations in plasma AAs after supplementation were associated with patients’ daily nitrogen intakes, similar to those found at baseline. This means that nitrogen from supplemented EAA replaced an equivalent amount of habitual diet nitrogen, nevertheless providing a more efficient metabolic activity. To support this, the increase in plasma AAs occurred without increases in urea production/accumulation, notwithstanding the worsened renal dysfunction. 

The altered plasma AAs may exert favorable effects on the study patients, including increased liver supply of glucogenic amino acids, mainly glutamine, and increased renal glutamine availability, leading to ammonia excretion and bicarbonate reabsorption. Improved muscle glutamine generation reduces muscle metabolic acidosis by buffering ammonia from protein over degradation [119]. Increased glutamine may be taken up by enterocytes and immune cells. This is mainly of particular importance in patients with end stage renal disease, which is associated with an activated immune system and systemic inflammation [120]. Although enterocytes mainly obtain aspartic acid, asparagine and serine from the diet, it cannot be excluded that in a context of high intestinal mucosa demand, part of the circulating levels was taken up by enterocytes. Very importantly, asparagine infusion could repair TJ damage by inducing proliferation of intraepithelial lymphocytes and alleviation of intestinal inflammation [121]. Increased serine levels may suggest a reduction in utilization of the muscle glycolytic pathway and/or augmented renal release, as the kidney is normally the organ that produces 100% serine [122].

Increased plasma threonine may reflect an improved intestinal bacteria production. Indeed, in normal adults, microbial contribution to plasma threonine is 21 to 44.7 mg/kg/d [96].

After 6 months of EAA supplementation, the worsening of the phe/tyr ratio was probably due to reduced hydroxylation activity of phenylalanine [122] by the enzyme phenylalanine hydroxylase, an enzyme present in the liver, pancreas and kidney [123]. The renal patients have, in both body [124] and kidney [125,126], reduced phenylalanine hydroxylation activity. We cannot exclude that dietary and supplemented phenylalanine intake by patients contributed to the amplification of the phe/tyr ratio in a context of impaired phenylalanine hydroxylation.

Aspartic acid is one of the most important fuels and sources of aerobic ATP in mammalian enterocytes [127]. Dietary supplementation of aspartate enhances intestinal energy status and integrity [127]. Lacking enterocyte energy leads to intestinal damage [128]. Conversion of aspartate to ornithine increases synthesis of polyamines [129], substances that regulate intestinal epithelial growth and proliferation, and its transformation to arginine ensures the direct protection of the intestine, as arginine-produced Nitric Oxide (NO) protects the intestine from blood-born and luminal toxins. 

The ingested amount of EAAs was probably not sufficient to drive a net muscle protein synthesis. This may explain the lack of improvement of skeletal muscle mass. This is in line with a previous study [111] documenting that the reduction in the rate of contractile protein synthesis may occur despite the body protein turnover being similar to that in controls. 

The maintenance of normal extramuscular protein turnover may also explain the absence in our study of increases in blood urea levels from increased plasma AAs despite the worsening of renal filtration dysfunction. 

### 6.4. Patients’ Characteristics at Baseline and after 6 Months of EAA Supplementation

At baseline, the co-presence of deteriorated muscle mass and normal body weight was due to patients’ water retention. In CKD, inflammation/oxidative stress [10,130,131,132]; production of catabolic hormones such as glucagon, catecholamines and cortisol [111,133]; and metabolic acidosis [108,134] are all factors that, variously combined, can induce an excess of skeletal muscle protein degradation. In addition, low IGF-1 bioavailability following increased serum IGF-1 binding protein [135,136] contributes to reduced muscle protein synthesis.

Lack of erythropoietin synthesis, inhibition of erythropoietin in bone marrow by cytokine overproduction [137,138] and bone marrow reduced sensitivity to erythropoietin under inflammatory conditions [137,138] can explain patient anemia. Of note, angiotensin II is now considered a cytokine that regulates inflammation and fibrosis [139]. Importantly, uremic toxins via impaired erythropoietin synthesis contribute to the development of anemia [140].

Compared to controls, the increased plasma levels of aspartic acid may be due to an increased conversion of oxalacetate via the transamination process, leading to reduced oxalacetate availability in the citric acid cycle. This may lead to mitochondria dysfunction [111].

Increased histidine levels may derive from glutamine that is consequently shifted away from the citric acid cycle. In addition/alternatively, histidine may originate from histidine-peptides carnosine, anserine, balanin used for buffering intracellular protons, neutralization for reactive oxygen species and Ca^2+^ regulation [112]. 

Increased aspartate and histidine levels could suggest the presence of muscle mitochondria dysfunction, in line with a previous study documenting a reduced synthesis rate of mitochondrial protein and impaired activity of cytochrome c-oxidase and citrate synthase in CKD patients [111].

The patients’ protein intakes revealed a partial adherence to the prescribed diet (0.6–0.7 g/kg/d). The lack of adherence to diet protein prescriptions to CKD patients is a common concern [141,142,143,144,145].

After 6 months of EAA supplementation, worsened renal dysfunction was associated with improved intestinal barrier dysfunction and changes in plasma amino acids, with favorable implications for gut barrier homeostasis as discussed above. The discrepancy between the worsening of renal dysfunction and the improvement of intestinal epithelial barrier dysfunctions might indicate that noxious agents other than enterotoxins affected kidney (dys-)function. Among these agents, an overload of nitrogen-wasting product should not be annoverated, as bun levels were virtually unchanged, notwithstanding the increased circulating total amino acid levels. This means that in the study patients, under overnight fasting, the NEAAs released from muscles were taken up by extramuscular districts. 

## 7. Was the Study Mixture Appropriate to the Study Patients?

The mixture used in the current study included methionine, tryptophan and phenylalanine, i.e., three AAs that are theoretically inappropriate to patients with CKD for the reasons discussed above.

However, given the improvement in intestinal integrity, the supplementation of these EAAs was shown to not be detrimental. The safety of their ingestion may be due to the small amounts of tryptophan, methionine and phenylalanine contained in the two sachets of the EAA mixture (45%, 20.4%, 25%, respectively, of their contents in 100 gr of lean beef meat [146]. Supporting the safety of supplemented EAAs, the plasma levels of tryptophan and phenylalanine remained virtually unchanged after 6 months of EAA supplementation. To the best of our knowledge, this is the first study documenting the safety of small amounts of supplemented tryptophan and phenylalanine in CKD patients. For methionine, its 159% increase in plasma levels after EAA supplementation should not be attributed to the small amount of ingested methionine but to altered metabolism, as discussed above.

## 8. Limits of the Study

The identification of gut-altered microorganisms in the study patients and the modification towards possible increases in healthier bacterial populations by means of supplemented EAAs would have provided valuable information for future specific studies about the possibility of improving gut microorganisms with supplemented EAAs.

Another limit of the study was the low patient adherence to the prescribed diet (0.6 g protein/kg/day). Patient adherence to the prescribed protein and supplemented EAAs would probably have reduced the levels of blood and intestinal urea nitrogen impacting intestinal function and may have provided information about whether 0.6 g/kg/d + EAAs could be safe for the protein turnover of TJs. The study has not investigated the renal tubular damage in either pre- or post-EAA supplementation phases. An influence of EAAs on tubuli metabolism cannot be excluded, given the high mitochondrial activity of the tubuli [147].

A larger number of treated and control CKD patients would have reinforced the discussion.

A determination of fecal calprotectin and Zo at 3 months from the onset of EAA supplementation would have provided more information about their changes over time.

Nevertheless, for the statistical tests we used, the type I error is not affected by unequal group sizes, and, although it was small, the number was adequate to highlight differences (i) in fecal calprotectin and Zo levels, (ii) in plasma amino acids between CKD patients and controls and (iii) among CKD patients between 6 months supplementation and baseline values. Moreover, we believe that the very selective exclusion criteria adopted in this study, including cigarette smoking, could better show the pathophysiological interrelations among kidney dysfunction, gut dysbiosis and their partial changes associated with the supplemented mixture.

## 9. Conclusions

The study shows that a supplementation of a mixture containing EAAs and mitochondrial intermediates might be associated with improvements in gut inflammation and permeability in elderly subjects with CKD. Future research addressing the chance of improving intestinal damage by nutraceutical agents, including combined EAAs and intermediates of the TCA cycle, must be carried out before drawing any useful information for clinical practice.

## Figures and Tables

**Table 1:** Composition of the mixture used in the current study.

Ingredients	mg/Sachet
**Amino acids**	
L-leucine *	1200
L-lysine HCl *	1129.23
L-threonine *	700
L-isoleucine *	600
L-valine *	600
L-fenilalanine *	100
L-tryptophan * L-methionine *	50 50
L-cysteine L-histidine	150 150
**Intermediates of TCA**	
Citric acid	409
Succinic acid	102.50
Malic acid	102.50
**Vitamines**	
Beta carotene	10
Pyridoxine hydrocloride	1.03
Thiamine hydrocloride	0.90
**Others**	
Polysorbate 80 Flavouring Sucralose Acesulfame K	237.50 214.94 24 18

* Essential amino acids.

**Table 3:** Baseline measures associated with intestinal mucosa inflammation (calprotectin, µg/g) and permeability (zonulin, ng/ml) of controls (CTR) and Chronic Kidney Disease (CKD) patients.

Variables	CTR	CKD	*p*
Calprotectin	30.25 ± 27.62	95.60 ± 53.26	0.005 ^
Zonulin	54.96 ± 32.73	219.38 ± 171.50	0.001 ^

Reported *p*-values are from Mann-Whitney U-test. ^: significance confirmed controlling for the False detection rate at 5% (Benjamini Hockberg method).

**Table 4:** Baseline plasma Amino Acid (AA, µmol/l) profile in controls (CTR) and Chronic Kidney Disease (CKD) patients.

Amino Acids	CTR	CKD	*p*
Aspartic acid	4.84 ± 1.78	14.39 ± 5.50	0.0005 ^
Glutamic acid	159.49 ± 21.75	179.92 ± 46.72	0.45
Asparagine	10.51 ± 2.12	19.57 ± 15.65	0.034
Serine	31.60 ± 6.77	40.41 ± 7.35	0.016
Glutamine	147.22 ± 27.46	229.41 ± 131.79	0.18
Histidine	22.24 ±3.72	63.13 ± 31.61	0.002 ^
Glycine	123.63 ± 34.81	176.66 ± 66.84	0.043
Threonine	65.18 ± 14.40	89.34 ± 29.27	0.13
Alanine	348.94 ± 84.88	316.25 ± 39.76	0.46
Arginine	95.73 ± 15.79	158.22 ± 61.73	0.08
Tyrosine	54.69 ± 12.41	47.66 ± 7.48	0.32
Cysteine	207.54 ± 56.71	208.18 ± 54.18	0.92
Valine	172.90 ± 19.25	168.03 ± 35.51	0.92
Methionine	19.12 ± 3.48	21.40 ± 5.23	0.45
Tryptophan	34.53 ± 4.95	48.61 ± 21.17	0.25
Phenylalanine	45.11 ± 4.76	48.63 ± 6.45	0.21
Isoleucine	47.62 ± 8.39	50.46 ± 11.84	0.36
Leucine	90.32 ± 15.38	88.59 ± 20.23	0.99
Lysine	125.23 ± 15.70	134.89 ± 17.86	0.25
Proline	196.71 ± 63.87	233.29 ± 125.77	0.73
Total AA	2003.15 ± 239.91	2337.05 ± 419.62	0.11
Essential AA	622.25 ± 66.72	713.08 ± 140.98	0.36
Non Essential AA	1380.90 ± 218.82	1623.97 ± 317.28	0.11
Branched Chain AA	310.85 ± 40.79	307.08 ± 66.30	0.92
Phenylalanine / Tyrosine ratio	0.85 ± 0.13	1.04 ± 0.17	0.010 ^

Reported *p*-values are from Mann-Whitney U-test. ^: significance confirmed controlling for the False detection rate at 5% (Benjamini Hockberg method).

**Table 5:** Measures associated with intestinal mucosa inflammation (calprotectin, µg/g) and permeability (zonulin, ng/ml) of Chronic Kidney Disease (CKD) patients at baseline and after 6 months of AA supplementation.

Variables	Baseline	After 6 months AA	*p*
Calprotectin	95.60 ± 53.26	37.92 ± 22.04	0.008^
Zonulin	219.4 ± 171.5	70.5 ± 35.9	0.05

Reported *p*-values are from Wilcoxon signed rank test. ^: significance confirmed controlling for the False detection rate at 5% (Benjamini Hockberg method).

**Table 6:** Plasma Amino Acid (AA, µmol/l) profile in Chronic Kidney Disease (CKD) patients at baseline and after 6 months of AA supplementation.

Amino Acids	Baseline	After 6 Months AA	*p*
Aspartic acid	14.39 ± 5.50	31.75 ± 10.32	0.016
Glutamic acid	179.9 ± 46.7	112.3 ± 36.5	0.016
Asparagine	19.57 ± 15.65	61.72 ± 16.60	0.016
Serine	40.41 ± 7.35	68.36 ± 16.39	0.016
Glutamine	229.4 ± 131.8	429.0 ± 119.5	0.008
Histidine	63.13 ± 31.61	91.53 ± 26.89	0.20
Glycine	176.7 ± 66.8	218.2 ± 58.4	0.55
Threonine	89.34 ± 29.27	130.79 ± 65.22	0.05
Alanine	316.3 ± 39.8	410.0 ± 78.2	0.023
Arginine	158.2 ± 61.7	222.5 ± 59.1	0.08
Tyrosine	47.66 ± 7.48	47.56 ± 10.16	1.00
Cysteine	208.2 ± 54.2	233.6 ± 76.6	0.64
Valine	168.0 ± 35.5	145.0 ± 56.1	0.46
Methionine	21.40 ± 5.23	55.53 ± 10.09	0.008
Tryptophan	48.61 ± 21.17	50.86 ± 15.54	0.95
Phenylalanine	48.63 ± 6.45	58.66 ± 16.85	0.15
Isoleucine	50.46 ± 11.84	54.54 ± 17.94	0.55
Leucine	88.59 ± 20.23	92.09 ± 31.78	0.84
Lysine	134.9 ± 17.9	147.3 ± 43.4	0.84
Proline	233.3 ± 125.8	300.7 ± 180.3	0.38
Total AA	2337 ± 420	2962 ± 719	0.11
Essential AA	713.1 ± 141.0	826.3 ± 257.7	0.38
Non Essential AA	1624 ± 317	2136 ± 478	0.05
Branched Chain AA	307.1 ± 66.3	291.7 ± 104.7	0.55
Phenylalanine/Tyrosine ratio	1.04 ± 0.17	1.23 ± 0.22	0.05

Reported *p*-values are from Wilcoxon signed rank test.

**Table 7:** Association (Spearman r) between Δ Zonulin (Zonulin after 6 months AA supplementation–baseline Zonulin, second column) and Δ Calprotectin (Calprotectin after 6 months AA supplementation—baseline Calprotectin, third column) and Δ plasma Amino Acid (AA) profile in Chronic Kidney Disease.

	Correlation with Δ Zonulin Spearman r (*p* Value)	Correlation with Δ Calprotectin Spearman r (*p* Value)
Δ Aspartic	0.357 (0.39)	0.095 (0.84)
Δ Glutamic acid	−0.429 (0.30)	−0.476 (0.24)
Δ Asparagine	0.238 (0.58)	−0.024 (0.98)
Δ Serine	0.024 (0.98)	−0.405 (0.33)
Δ Glutamine	0.619 (0.11)	0.429 (0.30)
Δ Histidine	0.500 (0.22)	0.310 (0.46)
Δ Glycine	0.262 (0.54)	−0.024 (0.98)
Δ Threonine	0.571 (0.15)	0.095 (0.84)
Δ Alanine	−0.357 (0.39)	0.024 (0.98)
Δ Arginine	0.357 (0.39)	−0.024 (0.98)
Δ Tyrosine	0.643 (0.10)	0.357 (0.39)
Δ Cysteine	0.500 (0.22)	0.024 (0.98)
Δ Valine	0.714 (0.05)	0.405 (0.33)
Δ Methionine	0.429 (0.30)	0.262 (0.54)
Δ Tryptophan	0.643 (0.10)	0.310 (0.46)
Δ Phenylalanine	0.357 (0.39)	0.095 (0.84)
Δ Isoleucine	0.452 (0.27)	−0.095 (0.84)
Δ Leucine	0.619 (0.11)	0.333 (0.43)
Δ Lysine	0.643 (0.10)	0.595 (0.13)
Δ Proline	−0.667 (0.08)	−0.643 (0.10)
Δ Total AA	0.190 (0.66)	−0.048 (0.93)
Δ Essential AA	0.857 (0.024)	0.262 (0.54)
Δ Non Essential AA	0.190 (0.66)	−0.048 (0.93)
Δ Branched Chain AA	0.571 (0.15)	0.238 (0.58)

**Table 8:** Glutamine/(BCAA + aspartic acid + asparagine + glutamate) and Alanine/(BCAA + aspartic acid + asparagine + glutamate) ratio in CKD patients at baseline and after 6 months of AA supplementation.

Variables	Baseline	After 6 months AA	*p* Value
Gln/(BCAA+Asp+Asn+Glu)	0.43 ± 0.23	0.91 ± 0.29	0.008^
Ala/(BCAA+Asp+Asn+Glu)	0.61 ± 0.08	0.88 ± 0.25	0.008^

BCAA: Branched Chain Amino Acids. ^: significance confirmed controlling for the False detection rate at 5% (Benjamini Hockberg method).

**Table 9:** Blood Urea Nitrogen (BUN), BUN / Total Amino Acids (TAAs) BUN/non Essential AA (non EAAs) ratio in CKD patients at baseline and after 6 months of AA supplementation.

Variable	Baseline	After 6 Months AA	*p* Value
BUN (mg/dl)	43.86 ± 16.09	50.33 ± 9.69	0.23
BUN/(TAAs)	0.020 ± 0.012	0.018 ± 0.006	0.84
BUN/(Non EAAs)	0.030 ± 0.018	0.025 ± 0.009	0.55

## Data Availability

Data available on request due to restrictions eg privacy or ethical. The data presented in this study are available on request from the corresponding author.
